# Comparative Analysis of Gut Microbiota from Rats Induced by Se Deficiency and T-2 Toxin

**DOI:** 10.3390/nu15245027

**Published:** 2023-12-07

**Authors:** Yifan Wu, Yi Gong, Yu Zhang, Shujin Li, Chaowei Wang, Yuequan Yuan, Xi Lv, Yanli Liu, Feihong Chen, Sijie Chen, Feiyu Zhang, Xiong Guo, Xi Wang, Yujie Ning, Hongmou Zhao

**Affiliations:** 1 Department of Occupational and Environmental Health, School of Public Health, Xi’an Jiaotong University Health Science Center, Xi’an 710061, China; 3122315010wyf@stu.xjtu.edu.cn (Y.W.); zhangyu0925@stu.xjtu.edu.cn (Y.Z.); lyl0327@stu.xjtu.edu.cn (Y.L.); chenfeihong@stu.xjtu.edu.cn (F.C.); 2 MED-X Institute, Center for Immunological and Metabolic Diseases, The First Affiliated Hospital of Xi’an Jiaotong University, Xi’an 710061, China; gongyii2020@163.com; 3 Key Laboratory of Trace Elements and Endemic Diseases, School of Public Health, Xi’an Jiaotong University Health Science Center, National Health and Family Planning Commission, Xi’an 710061, China; 3122315054@stu.xjtu.edu.cn (S.L.); 3122315031@stu.xjtu.edu.cn (C.W.); yyuequan_@stu.xjtu.edu.cn (Y.Y.); lvxi0505@stu.xjtu.edu.cn (X.L.); chensijie@stu.xjtu.edu.cn (S.C.); zhangfeuyu77@stu.xjtu.edu.cn (F.Z.); guox@xjtu.edu.cn (X.G.); 4 Clinical Research Center for Endemic Disease of Shaanxi Province, The Second Affiliated Hospital of Xi’an Jiaotong University, No.157 Xi Wu Road, Xi’an 710004, China; 5 Key Laboratory for Disease Prevention and Control and Health Promotion of Shaanxi Province, Xi’an 710061, China; 6 Foot and Ankle Surgery Department, Honghui Hospital of Xi’an Jiaotong University, Xi’an 710001, China

**Keywords:** Kashin–Beck disease, T-2 toxin, selenium deficiency, gut microbiota, 16S rDNA gene sequencing

## Abstract

The aim of this study was to analyze the differences in gut microbiota between selenium deficiency and T-2 toxin intervention rats. Knee joint and fecal samples of rats were collected. The pathological characteristics of knee cartilage were observed by safranin O/fast green staining. DNA was extracted from fecal samples for PCR amplification, and 16S rDNA sequencing was performed to compare the gut microbiota of rats. At the phylum level, *Firmicutes* (81.39% vs. 77.06%) and *Bacteroidetes* (11.11% vs. 14.85%) were dominant in the Se-deficient (SD) group and T-2 exposure (T-2) groups. At the genus level, the relative abundance of *Ruminococcus_1* (12.62%) and *Ruminococcaceae_UCG-005* (10.31%) in the SD group were higher. In the T-2 group, the relative abundance of *Lactobacillus* (11.71%) and *Ruminococcaceae_UCG-005* (9.26%) were higher. At the species level, the high-quality bacteria in the SD group was *Ruminococcus_1_unclassified*, and *Ruminococcaceae_UCG-005_unclassified* in the T-2 group. *Lactobacillus_sp__L_YJ* and *Lactobacillus_crispatus* were the most significant biomarkers in the T-2 group. This study analyzed the different compositions of gut microbiota in rats induced by selenium deficiency and T-2 toxin, and revealed the changes in gut microbiota, so as to provide a certain basis for promoting the study of the pathogenesis of Kashin–Beck disease (KBD).

## 1. Introduction

Kashin–Beck disease (KBD) is a chronic, degenerative and malformed osteoarticular disease that mainly affects the epiphyseal plate cartilage and articular cartilage in children during the growth and development period [1]. Many etiological studies have shown that environmental selenium deficiency and T-2 toxin in grains could be important causes of KBD [2]. Previous studies found that the selenium content in food, drinking water and patients in KBD endemic areas was significantly lower than that in non-endemic areas [3,4]. In addition, the epidemiological survey showed that the distribution of KBD in China was an oblique band from northeast to southwest, which was highly coincident with the distribution of Se deficiency in China [5]. T-2 toxin is a tricothecene toxin produced by a variety of fusarium species [6]. In previous studies, it was suggested that T-2 toxin could cause inflammatory damage of chondrocytes and upregulate the expression of KBD susceptibility genes in chondrocytes [7,8]. The conclusions of the previous studies have proved that the imbalance of selenium nutrition caused by low selenium in the external environment and T-2 toxin poisoning in grain are likely to be important causes of the etiology and pathogenesis of KBD [9].

Gut microbiota is a microbial community inhabiting the human gut and distributed throughout the gastrointestinal tract, which is a dynamic ecosystem. The entire gut microbiota is composed of more than 1000 species of bacteria, as well as fungi, viruses, phages, parasites and archaea [10]. Under normal conditions, different types of gut microbiota in the human body exist stably in accordance with a certain proportion and quantity, which are both interdependent and restricted to each other. *Firmicutes* and *Bacteroidetes* are the most typical bacterial phyla in the healthy gut microbiota, followed by *Actinobacteria*, *Proteobacteria* and *Fusobacteria* [11,12]. At the genus level, the most typical bacterial genera are *Bacteroides*, *Faecalibacterium* and *Bifidobacterium*. The bacteria of each classification exist dynamically and stably [13]. When stimulated by the internal and external environment, the species, quantity, proportion, and metabolites of each bacterial group will change [14]. Gut microbiota plays an important role in the regulation of various diseases, including diabetes, osteoarthritis, hypertension, chronic obstructive pulmonary disease (COPD), and obesity [15,16,17,18,19,20,21]. The influence of gut microbiota on the above diseases is achieved through the comprehensive effect of genetic and environmental factors [22]. Relevant studies have confirmed that gut microbiota can participate in host physiological activities through its metabolites, and can migrate to subchondral bone marrow and deep chondrocytes through systemic circulation to cause cellular inflammation, thereby inducing changes in cartilage tissue and causing osteochondral diseases [23]. It has also been shown that the cartilage–gut–microbiome axis plays a role in chondrocyte differentiation, apoptosis, and injury mechanism by regulating the levels of metabolites [24]. The concept of the cartilage–gut–microbiome axis provides new ideas for the etiology and treatment of bone and cartilage diseases, such as KBD and osteoarthritis. Previously, we found a comprehensive landscape of the gut microbiota in KBD patients, and compared the gut microbiota of osteoarthritis (OA) and KBD patients, which provided strong evidence for the correlation between gut microbiota and KBD.

As the key suspected causes of KBD, mycotoxin contamination in grain and environmental Se deficiency can affect the human body through the dietary channel [1,25,26]. Studies have shown that mycotoxins in grain are absorbed into the human body mainly in the form of glucose covalent conjugates of mycotoxins, and can reach the human intestine in intact form for hydrolysis and release, causing abnormal changes in gut microbiota [27]. In addition, selenium can increase the diversity of gut microbiota in mice and participate in the composition of gut microbiota, and it also can be enriched by the gut microbiota [28,29]. These results suggest that the changes in gut microbiota in KBD patients may be closely related to the above two environmental risk factors of KBD.

In this study, to further explore whether the gut microbiota of KBD patients is altered due to Se deficiency and T-2 toxin and its relationship with cartilage damage, we established the environment risk factors rat models of KBD induced by Se deficiency and T-2 toxin with an 8-week and 4-week intervention, respectively, and the characteristics and differences of gut microbiota after intervention in rats were analyzed and compared. This study will provide an important scientific basis for clarifying that T-2 toxin and/or Se deficiency may cause chondrocytes damage and metabolic disorders in KBD through the cartilage–gut–microbiome axis. 

## 2. Materials and Methods

### 2.1. Model Construction and Sample Collection

A total of 24 weaned Sprague Dawley rats (3-week-old, 30–50 g) were provided by the Experimental Animal Center of Xi’an Jiaotong University. The study protocol was approved by the Animal Ethics Committee of Xi’an Jiaotong University (No. 2018-206). The rats were reared in specific pathogen free (SPF) environment. The 24 rats were randomly divided into three groups: the normal control group (NC) was fed with control diet (selenium content: 0.18 ppm), the Se-deficient group (SD) was fed with Se-deficient diet (selenium content: 0.02 ppm), the T-2 exposure group (T-2) which fed with control diet were given T-2 toxin (200 ng/g·BW/day) by gavage [30], while the NC group and SD groups were given the same volume of 0.9% normal saline by gavage. All feeds of rats were purchased from Nantong Talofi Feed Company, Nantong, Jiangsu province, China. For intragastric administration: after the rats were fixed, the needle used for intragastric administration was inserted from the corner of the mouth, pushed gently against the roof of the mouth, and there was a feeling of hollow after entering the esophagus. Then, the needle was slowly inserted along the posterior wall of the pharynx into the esophagus, and the solution could be injected only when there was no air countercurrent in the syringe. To prevent the rats from failing to adapt to the T-2 toxin, in the T-2 group, the gavage treatment was performed at the age of 8 weeks and ended at 12 weeks, lasting a total of 4 weeks. Since the construction of the Se-deficient rat models need to last longer, the intervention of Se-deficient diet began at the age of 4 weeks and ended at 12 weeks, lasting a total of 8 weeks. The rats were fed in the mesh floor cages with 2 cages per group and 4 rats per cage, and all animals had free access to distilled water at all time. The weight of the rats was measured and recorded at a fixed time each week. During the experiment, we did not find that the food intake of rats was affected by the intervention regimen. The rats treated by Se-deficient diet had poor appetite and increased drinking water, and the rats treated by T-2 toxin were sluggish, less active, and more serious hair removal. By analyzing the weight records, it was found that the growth of rats in each group was in line with the normal development rate, and there was no statistical difference in each node. At the age of 12 weeks, fresh feces of the rats were collected with sterilized tweezers, and about 1 g (3–5 pellets) of feces were collected from each rat, of which 1–2 pellets were used for analysis and the rest for backup, all stored in the sterile EP tubes at −80 °C for follow-up experiments. At 24 h after the last gavage, the rats were anesthetized by intraperitoneal injection of 10% chloral hydrate (0.5 mL/100 g·BW). The rats were sacrificed by blood sampling from the abdominal aorta. After the rats were killed, we detected the serum selenium content of rats by hydride generation atomic fluorescence spectrometry (HG-AFS), and the results showed that the serum selenium content of rats in the SD group was significantly lower than that in the NC group, indicating that the Se-deficient model was successfully constructed. Relevant research results have been published [31]. During the dissection of the rats, the fur and muscle were stripped with scalpel, the knee joints on both sides of the rats were removed with surgical scissors. After carefully separating the connective tissue, the knee joints of rats were placed in the 50 mL centrifuge tubes with 4% paraformaldehyde fixing solution and stored at room temperature away from light for later use.

### 2.2. T-2 Toxin Solution Preparation

An amount of 5 mg T-2 toxin crystals (CAS: 21259-20-1, purchased from Beijing Bairingwei Technology Co., LTD, Beijing, China) were collected in a bottle, then 2 mL absolute ethanol was added, shaken and blended using a vortex mixer, and the liquid was sucked into a 250 mL beaker. The procedure was repeated three times to ensure the complete dissolution of T-2 toxin crystals on the bottom and wall of the bottle. The 0.9% saline was used to dilute the solution in the beaker, and the T-2 toxin solution with a concentration of 20 μg/mL was obtained. The solution was divided into small aliquots and frozen at −20 °C for subsequent experiment. Before each use, the solution was dissolved at room temperature and sonicated to homogenize the solute.

### 2.3. Safranin O/Fast Green Staining

The fixed knee joints samples of rats were decalcified with 10% (*w*/*v*) ethylenediaminete traacetic acid (EDTA) for 4 weeks. The decalcified samples were cut to appropriate size with a surgical blade, naturally dried and then subjected to ethanol gradient dehydration, xylene transparency, and paraffin embedding. The paraffin blocks were cut into serial sections with a thickness of 4–7 µm, mounted on slides, baked at 60 °C and stored at room temperature. Sections of the knee joint were deparaffinized with xylene, hydrated with gradient ethanol, and washed with tap water. The sections were stained with hematoxylin dye for 5 min, washed with tap water to remove excess dye, and rapidly differentiated in 1% hydrochloric acid solution for several seconds. The step of fast green staining took 10 min, and the excess dye was washed off with water. Then, the slices were placed in safranin O staining solution for 45 s, washed with water and slightly soaked in differentiation solution. Finally, the sections were rapidly dehydrated in absolute ethanol, transparent through xylene, and then sealed with neutral gum for use. The stained cartilage sections were observed and the images were collected by automatic digital slicing scanner (Pannoramic desk, purchased from 3Dhistech, Budapest, Hungary).

### 2.4. DNA Extraction of Gut Microbiota

Fecal samples were collected from rats at 12 weeks of age, and the total DNA of the gut microbiota in the fecal samples was extracted according to the instructions of the E.Z.N.A.^®^Stool DNA Kit (D4015-02, Omega, Inc., Norcross, GA, USA). It has been demonstrated that the reagent, which was designed to extract DNA from trace amounts of sample, is efficient for preparing the DNA of most bacteria. Unused swabs processed through DNA extraction were utilized as sample blanks, and the absence of any DNA amplicons was confirmed in sample blanks. The quality of DNA extraction was detected by agarose gel electrophoresis, and DNA was quantified using a UV spectrophotometer. According to a modified version of the manufacturer’s (Omega) instructions, the total DNA was eluted in 50 L of elution buffer and kept at −80 °C until it was measured in a PCR by LC-BIO TECHNOLOGIES (HANGZHOU) CO., LTD., Hang Zhou, Zhejiang Province, China.

### 2.5. 16S rDNA Gene Sequencing

The V3–V4 variable region of 16S rDNA gene was selected as the target region for PCR amplification. The upstream primer was 341F (5’-CCTACGGGNGGCWGCAG-3’) and the downstream primer was 805R (5’-GACTACHVGGGTATCTAATCC-3’). The 5′ ends of each primer were marked with a unique barcode for each sample, and the universal primers were sequenced. PCR amplification was performed after a total volume of 25 μL of the reaction mixture including 25 ng of template DNA, 12.5 μL PCR Premix, 2.5 μL of each primer, and PCR-grade water which used to adjust the volume was prepared. PCR reaction conditions were as follows: first, predenaturation at 98 °C for 30 s; and then there were 32 cycles of denaturation at 98 °C for 10 s, annealing at 54 °C for 30 s, and extension at 72 °C for 45 s. The final extension lasted for 10 min at 72 ° C. In order to exclude the possibility of false-positive PCR results as a negative control, ultrapure water was utilized instead of sample solution throughout the DNA extraction procedure. The products of PCR were purified using AMPure XT Beads (Beckman Coulter Genomics, Danvers, MA, USA) and quantified with Qubit (Invitrogen, Carlsbad, CA, USA). The amplicon pool was used for the sequencing process, and an Agilent 2100 Bioanalyzer (Agilent, Santa Clara, CA, USA) and the Illumina Library Quantification kit (Kapa Biosciences, Woburn, MA, USA), respectively, were used to measure the size and quantity of the amplicon libraries. On NovaSeq PE250 platform, the libraries were sequenced.

Samples were sequenced using the Illumina NovaSeq platform in accordance with the manufacturer’s specifications. Paired-end sequences were assigned based on the unique barcode of each sample, and the barcode and primer sequence were both truncated. Paired-end reads were merged using FLASH. To produce high-quality clean labels, the raw reads were quality filtered using fqtrim (v0.94) under specific filtering parameters. In addition, the chimeric sequences were filtered by Vsearch software (v2.3.4). After data deduplication, feature tables and feature sequences were obtained. The distances were determined for PCoA and LEfSe using the Bray–Curtis method. We used QIIME2 and R (v3.5.2) to calculate the Alpha diversity and Beta diversity and draw the pictures, respectively, reduced the number of sequences in some samples to a minimum by randomly extracting the same number of sequences, and classified bacteria using the relative abundance. The relative abundance of each sample was used to normalize the feature abundance according to SILVA classifier. Blast was used for sequence alignment, the SILVA and NT-16S databases were used for annotation of each representative sequence, and R (v3.5.2) was used for drawing pictures. LEfSe analysis was performed as the following steps: firstly, all characteristic species was detected using Kruskal–Wallis rank sum test, and species with significant differences were obtained by measuring species abundance differences among different groups. Secondly, the Wilcoxon rank sum test was used to detect all subspecies of the significantly different species obtained in the previous step to determine whether they converged to the same taxonomic level. Finally, linear discriminant analysis (LDA) was used to obtain the final differential species.

### 2.6. Statistical Analysis

We used SPSS 18.0 software for statistical analysis of the measured data and normality tests for continuous variables. If the data met the normal distribution, Student’s *t*-test and one-way analysis of variance (ANOVA) were used to evaluate the difference between the two groups, and the LSD-*t* test was further used for comparison between the two groups. Otherwise, the data were reanalyzed after transformation, or the rank-sum test was used for comparison analysis between groups. Spearman correlation analysis was used to explore the correlation between variables. *p*-value < 0.05 was considered statistically significant. Statistical plots were drawn using GraphPad Prism 8.0.

## 3. Results

### 3.1. Pathological Changes in the Knee Joint of the Rats 

**Figure 1:**
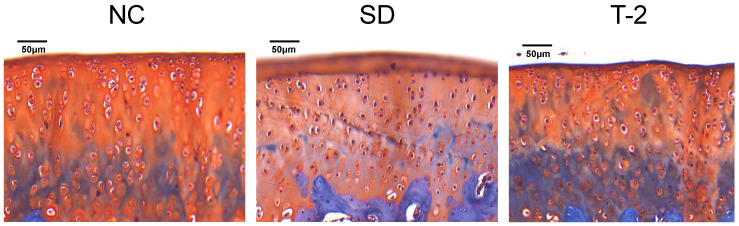
The articular cartilage of the rat knee joint stained with safranin fast green. NC, normal control group; SD, Se-deficient group; T-2, T-2 toxin exposure group.

In the NC group, the cartilage matrix was uniformly red, and the subchondral bone was blue-green, and the cartilage tissue was in sharp contrast with the bone tissue. The articular cartilage was smooth, and the chondrocytes were evenly distributed and arranged neatly. Compared with the NC group, the cartilage zone was thinner, the surface of cartilage appeared fissure, and the red part was significantly reduced in the SD and T-2 group. In addition, articular cartilage showed obvious wear and matrix loss (Figure 1).

### 3.2. Analysis of Gut Microbiota in Rats Exposed to Se Deficiency vs. Negative Control Rats

**Figure 2:**
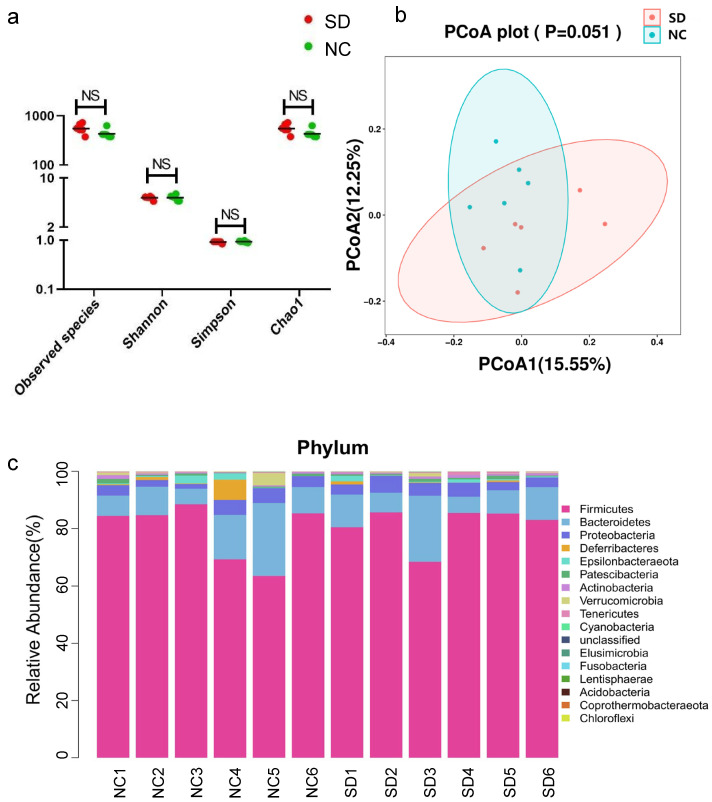
(**a**) Species diversity differences between the SD and NC groups were estimated by the observed species, Shannon, Simpson, and Chao1 indices. SD, the Se deficiency group; NC, the negative control group; (**b**) principal coordinate analysis (PCoA) of the gut microbiota based on the unweighted (ANOSIM, *p* = 0.051) UniFrac distance matrices for the SD and NC groups; (**c**) bar chart of species abundance at the phylum level in the SD group and NC group; *n* = 6 for the SD group and *n* = 6 for the NC group.

A total of 914,275 high-quality reads were obtained from 16S rDNA sequencing, with a median of 77,072.5 reads per sample (71,749 to 79,342). There were 8621 features, including 4029 in the SD group and 4592 in the NC group. The alpha diversity and beta diversity were compared between the SD and NC groups to evaluate the characteristics of gut microbiome in rats intervened by Se deficiency. The alpha diversity was used to assess species richness and evenness of microorganisms in individual sample. In this study, observed species and Chao 1 indices were used to reflect richness, while Shannon and Simpson indices could comprehensively reflect richness and evenness in samples. The results showed that there was no significant difference in Shannon, observed species, Simpson and Chao 1 indices between the SD group and the NC group, indicating that the richness and evenness of microbial communities in the two groups did not change significantly (Figure 2a). The beta diversity was used to measure the magnitude of similarity in microbial community composition between different samples. Principal co-ordinates analysis (PCoA) was used to observe the similarities and differences between groups, and to visualize the similarity or difference of the data. In this study, the PCoA results of both weighted (Figure S1a) and unweighted UniFrac (Figure 2b) showed that there was no significant difference in beta diversity between the SD group and the NC group (*p* > 0.05), indicating that there was no significant difference in the composition and distribution of microbial communities between the two groups. 

**Figure 3:**
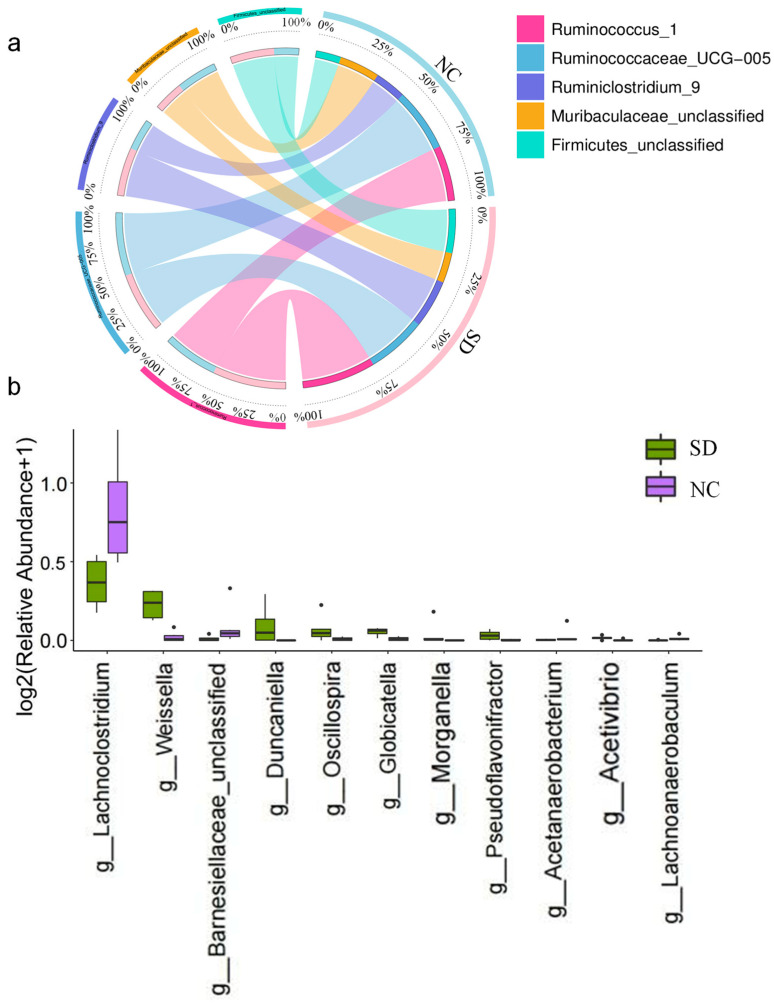
(**a**) Circos plot of species abundance at the genus level in the SD group and the NC group; SD, the Se deficiency group; NC, the negative control group; (**b**) Analysis of Species significant differences at the genus level in the SD group and the NC group. The black dot on each column indicates that one of the samples in the group has a higher abundance value than the other samples.

Microbial taxon division was performed to evaluate the relative proportions of dominant taxa at the genus level between the SD and NC groups. *Firmicutes* was the dominant phylum in the SD and NC groups, accounting for 81.39% and 79.29%, respectively. The proportion of *Bacteroidetes* in the SD group (11.11%) was lower than that in the NC group (12.12%). On the contrary, the proportion of *Proteobacteria* in SD group (4.17%) was higher than that in the NC group (3.61%) (Figure 2c). At the phylum level, *Firmicutes* was the dominant microbiome in the SD group. At the genus level, in the SD group, the relative abundance of *Ruminococcus_1* was increased and *Ruminococcaceae_UCG-005* was decreased. At the species level, the dominant microbiome of the two groups were *Ruminococcaceae_UCG-005_unclassified* (Figure 3a).

Linear discriminant analysis effect size (LEfSe) could identify two or more biomarkers and identify the microbiome with significant differences in abundance between groups. The threshold of LEfSe analysis was set as LDA value > 3, *p* < 0.05. In the comparison between the SD group and the NC group, the significant difference species with LDA value > 3 was *Lachnoclostridium* in the NC group, which was a biomarker with statistical differences between the two groups. At the genus level, *Lachnoclostridium*, *Weissella*, *Barnesiellaceae_unclassified* and *Duncaniella* had significant differences in relative abundance (Figure 3b).

In addition, species with the top 30 relative abundance at the genus level were selected. The correlation between these dominant bacterial groups was calculated based on their abundance using the SparCC method, and *p* values were obtained. The relationship pairs with the absolute value of correlation coefficient > 0.4 were selected from the results, and the correlation network diagram was drawn. Among the top 30 species of relative abundance at the genus level, there was significant positive correlation between *Ruminococcus_2* and *Ruminococcus_1*, followed by *Ruminococcus_1* and *Ruminococcaceae_UCG_014*. There was the strongest negative correlation between *Bacteroides* and *Ruminococcaceae_UCG_005* (Figure S1b). The COG database was used to annotate the gene function of the microbiota in samples, which can identify a variety of important functions, such as Na^+^/alanine symporter, Transposase (or an inactivated derivative) and maltose binding periplasmic protein periplasmic protein MalE (Figure S2).

### 3.3. Analysis of Gut Microbiota in Rats Exposed to T-2 Toxin vs. Negative Control Rats

**Figure 4:**
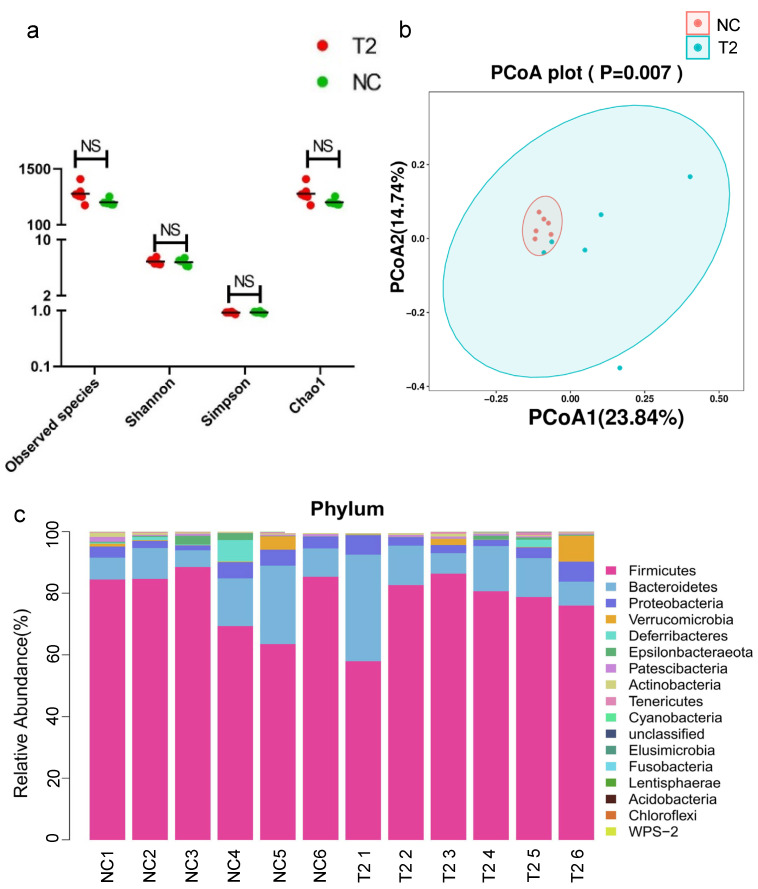
(**a**) Species diversity differences between the T-2 and NC groups were estimated by the observed species, Shannon, Simpson, and Chao1 indices. T-2, the T-2 toxin exposure group; NC, the negative control group; (**b**) principal coordinate analysis (PCoA) of the gut microbiota based on the unweighted (ANOSIM, *p* = 0.007) UniFrac distance matrices for the T-2 and NC groups; (**c**) bar chart of species abundance at the phylum level in the T-2 group and NC group; *n* = 6 for the T-2 group and *n* = 6 for the NC group.

A total of 884,350 high-quality 16S rDNA reads were obtained by sequencing, with a median of 74,370.5 reads per sample (ranging from 67,143 to 77,629). There were 9271 features, including 5272 in the T-2 group and 3999 in the NC group. The alpha diversity and the beta diversity were compared between the T-2 group and the NC group to evaluate the characteristics of gut microbiota in rats intervened by T-2 toxin. The results showed that there was no significant difference in Shannon, observed species, Simpson and Chao 1 indices between the T-2 group and the NC group, indicating that the richness and evenness of microbial community in the two groups did not change significantly (Figure 4a). In this study, the PCoA results of unweighted UniFrac showed that the beta diversity of the T-2 group and the NC group was significantly different (*p* < 0.05) (Figure 4b), while the PCoA results of weighted UniFrac were opposite (*p* > 0.05) (Figure S3a), suggesting that there were differences in the species of the microbiota between the two groups, but there may be no significant difference in species abundance. 

**Figure 5:**
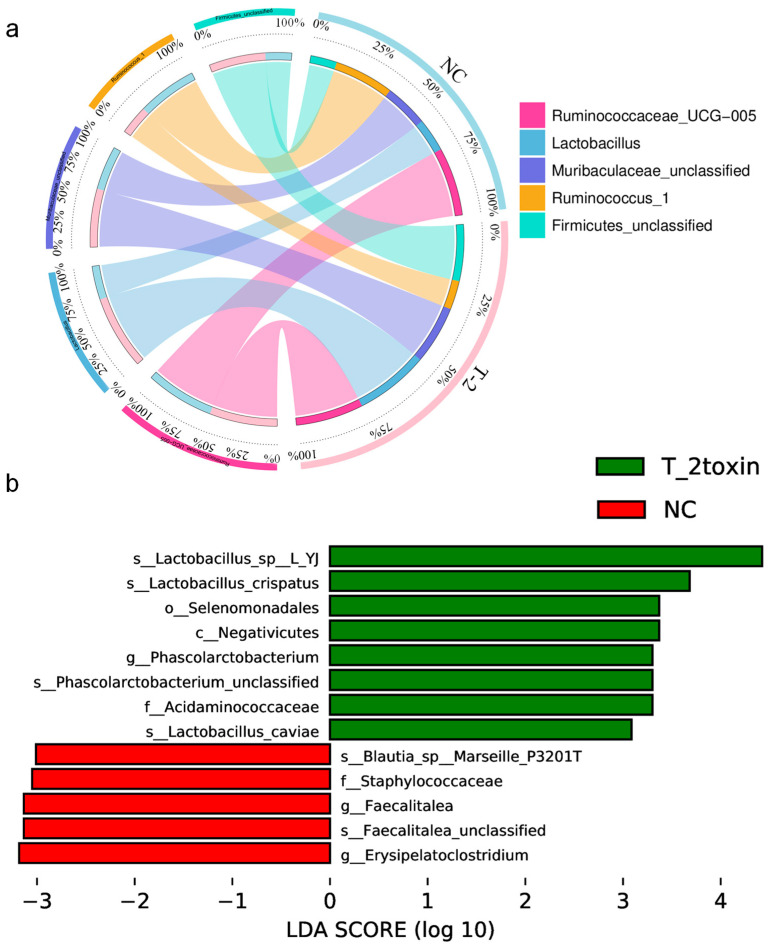
(**a**) Circos plot of species abundance at the genus level in the T-2 group and the NC group; T-2, the T-2 toxin exposure group; NC, the negative control group; (**b**) significantly different species with LDA > 3 in the linear discriminant analysis effect size (LEfSe); the threshold of LEfSe analysis was set as LDA > 3, *p* < 0.05.

To evaluate the relative proportions of dominant taxa at the genus level between the T-2 group and the NC group, microbial taxa were divided. *Firmicutes* was the dominant phylum in the T-2 group and the NC group, accounting for 77.06% and 79.30%, respectively. The proportions of *Bacteroidetes* and *Proteobacteria* in the T-2 group were both higher than those in the NC group, which were 14.85% vs. 12.12% and 3.95% vs. 3.61%, respectively (Figure 4c). At the phylum level, *Firmicutes* was the dominant microbiome in the T-2 group. At the genus level, the relative abundance of *Lactobacillus* in T-2 group was the highest, while *Ruminococcaceae_UCG-005* was the most dominant in the NC group. At the species level, the dominant bacteria of the two groups were *Ruminococcaceae_UCG-005_unclassified* (Figure 5a).

In the results with LDA value > 3, the significant difference species in the T-2 group were *Lactobacillus_sp_L_YJ* and *Lactobacillus_crispatus*. However, the NC group was characterized by *Erysipelatoclostridium* and *Faecalitalea* (Figure 5b). At the genus level, the species with significant differences in relative abundance were mainly *Phascolarctobacterium*, *Erysipelatoclostridium*, *Faecalitalea* and *Veillonella* (Figure S3b). Among the top 30 species in relative abundance at the genus level, *Lactobacillus* was positively correlated with *Christensenellaceae_R_7_group* and *Muribaculaceae_unclassified*. There was a negative correlation between *Bacteroides* and *Ruminococcaceae_UCG_005* (Figure S3c).

Several important functions were identified using the COG database, such as ATPase components of ABC transporters with duplicated ATPase domains and carbamoylphosphate synthase large subunit and guanylate kinase (Figure S4).

### 3.4. Analysis of Gut Microbiota in Rats Exposed to Se Deficiency vs. T-2 Toxin 

At the phylum level, *Firmicutes*, *Bacteroidetes*, *Proteobacteria*, *Actinobacteria* and *Verrucomicrobia* were the main phyla of gut microbiome in the SD and T-2 groups. *Firmicutes* had the highest relative abundance in the SD and T-2 groups, with SD group (81.39%) being higher than T-2 group (77.06%). The proportion of *Bacteroidetes* in SD group (11.11%) was lower than that in T-2 group (14.85%). On the contrary, the level of *Proteobacteria* was higher in SD group (4.17%) than in T-2 group (3.95%). In addition, *Actinobacteria* was the fourth most common bacteria in the SD group (0.70%), while it was lower in the T-2 group (0.50%). The level of *Verrucomicrobia* in the T-2 group was 1.81%, while it was only 0.36% in SD group (Table 1).

At the genus level, *Ruminococcus*, *Lactobacillus*, *Firmicutes_unclassified* and *Muribaculaceae_unclassified* were the main genus in the SD group and T-2 group. In the SD group, *Ruminococcus* was the most dominant bacteria, accounting for 12.62% of *Ruminococcus_1*, 10.31% of *Ruminococcaceae_UCG-005*, and 8.26% of *Ruminiclostridium_9*, respectively. In the T-2 group, *Lactobacillus* was the most abundant genus, accounting for 11.71%. The second was *Ruminococcaceae_UCG-005* (10.75%). The third most abundant genus was *Muribaculaceae_unclassified* (9.26%). Similarly, the proportion of *Firmicutes_unclassified* was the fourth in the SD group and the T-2 group, which was 7.55% and 8.95%, respectively, showing a higher level in the T-2 group (Table 1).

In addition, at the genus level, a total of 11 species with significant differences in relative abundance in the SD group were identified: *Weissella*, *Morganella*, *Lachnoanaerobaculum*, *Barnesiellaceae_unclassified*, *Globicatella*, *Acetivibrio*, *Duncaniella*, *Lachnoclostridium*, *Acetanaerobacterium*, *Oscillospira* and *Pseudoflavonifractor*. In the T-2 group, a total of 15 species with significant differences in relative abundance were identified: *Acetanaerobacterium*, *Hydrogenoanaerobacterium*, *Rikenella*, *Weissella*, *Veillonella*, *Phocea*, *Pseudoflavonifractor*, *Aerococcus*, *DTU014_unclassified*, *Ruminococcaceae_UCG-011*, *Eubacterium_xylanophilum_group*, *Acetovibrio*, *Phascolarctobacterium*, *Erysipelatoclostridium*, and *Faecalitalea*. Among them, *Weissella*, *Acetivibrio* and *Pseudoflavonifractor* were significantly increased in SD and T-2 groups, while *Acetanaerobacterium* was significantly decreased in both groups (Table 2A).

In the SD group, species with increased levels included *Morganella*, *Globicatella*, *Duncaniella* and *Oscillospira*; in the T-2 group, the levels of *Veillonella*, *Ruminococcaceae_UCG-011*, *Eubacterium_xylanophilum_group* and *Phascolarctobacterium* increased (Table 2B); in the SD group, the levels of *Lachnoanaerobaculum*, *Barnesiellaceae_unclassified* and *Lachnoclostridium* were decreased; in the T-2 group, the levels of *Hydroanaerobacterium*, *Rikenella*, *Phocea*, *Aerococcus*, *DTU014_unclassified*, *Erysipelatoclostridium* and *Faecalitalea* were decreased (Table 2C).

## 4. Discussion

Gut microbiota, composed of thousands of microbiota, strives to maintain the existence and function of intestinal mucosal barrier, participate in nutrient absorption and digestion, substance transport and metabolism, regulate human immunity, growth and development [32]. Studies on the changes in gut microbiota in osteochondral diseases have suggested that the gut microbiota of patients with osteochondral diseases is diverse, and there is a certain correlation between the pathogenesis of such diseases and gut microbiota [33]. Based on our previous study on the gut microbiota profile of KBD patients and the results of comparing the gut microbiota of OA and KBD patients, we constructed rat models of Se deficiency and T-2 toxin exposure, respectively, and conducted 16SrDNA sequencing to analyze gut microbiota, aiming to investigate whether gut microbiota affects the occurrence and development of KBD, hoping to provide inspiration for the prevention and treatment of KBD. 

In our previous study, the KBD patient was found to be characterized by elevated levels of *Fusobacteria* and *Bacteroidetes*. Consistent with the 16S rDNA analysis at the genus level, most of the differentially abundant species in KBD subjects belonged to *Prevotella* according to metagenomic sequencing. This indicates a change in the composition of the gut microbiota in patients with KBD. In the results of this study, *Firmicutes* were increased in the SD group compared with the NC group, while the level of *Bacteroidetes* were decreased. In contrast, the changes in the T-2 group showed a decrease in *Firmicutes* and an increase in *Bacteroidetes*. The changes in *Proteobacteria* were consistent in the SD and T-2 groups, which were higher than those in the NC group. In addition, the beta diversity of the T-2 group was significantly different from that of the NC group (*p* < 0.05), suggesting that the species of the T-2 group was different from that of the NC group. We can speculate that both Se deficiency and T-2 toxin exposure can dysregulate the composition of the gut microbiota in KBD, and the effects they play are different. 

As an important group of bacteria in the gut, *Bacteroidetes* are involved in a variety of metabolic activities and can decompose and utilize polysaccharides to help digest carbohydrates [34]. Among them, *Bacteroides* and *Prevotella* have attracted more attention from researchers. *Bacteroides* is involved in the degradation of polysaccharides and dietary fiber [35]. Studies have found that *Bacteroides* can stimulate the immune system, enhance the phagocytosis of macrophages, regulate the metabolism of the body, and induce the proliferation of probiotics to promote the health of the body [36]. *Bacteroides fragilis* has beneficial immunomodulatory effects on the body [37]. *Prevotella* can use polysaccharides to produce metabolites such as acetic acid and succinic acid [38]. Succinic acid can help maintain the body’s immunity and improve the health of the host [39]. Studies on the function of selenium point out that an adequate level of selenium is important for the initiation of immunity [40]. Se deficiency and inhibition of selenoproteins expression are associated with elevated levels of inflammatory cytokines in the gastrointestinal tract. Under Se deficiency conditions, innate and adaptive immune responses are impaired [41]. In addition, a study of T-2 toxin pointed out that T-2 toxin disrupts the gut microbiota by changing the relative abundance of species, genus, and phylum levels, and this change may be a direct effect of the toxin and its antibacterial properties, a toxic consequence of T-2 toxin on cells, or the release of antibacterial substances [42]. In this study, the level of *Bacteroidetes* in the SD group was decreased, indicating that the low selenium nutritional status caused the decrease in *Bacteroides* abundance, which led to the suppression of immune level, and then caused cartilage damage. However, the level of *Bacteroidetes* was increased in the T-2 group, which may be caused by the toxic characteristics of T-2 toxin. 

*Firmicutes* is the largest bacterial group in the gut, most of which are Gram-positive bacteria [43]. *Lactobacillus* is the most well-known probiotic, which is colonized in the human digestive tract and widely used in the food industry, medicine and health fields. It can participate in the metabolism of a variety of amino acids, help maintain the balance of gut microbiota, improve host immunity, and maintain the health of women’s vagina [44]. Studies have shown that *Lactobacillus* can ameliorate or limit inflammatory bone damage and joint dysfunction in patients with OA by reducing the expression of proinflammatory cytokines and cartilage damage [45]. Based on its anti-inflammatory effect, *Lactobacillus* has also been used as a probiotic treatment for OA for a long time [46]. In this study, compared with the NC group, the abundance of *Lactobacillus* in the T-2 group was significantly increased, and it was positively correlated with other bacteria such as *Christensenellaceae_R_7_group* and *Muribaculaceae_unclassified*. We speculated that the rise of *Lactobacillus* may be due to the lack of direct competition. These results indicated that T-2 toxin could not only unbalanced the gut microbiota of rats, but also regulate the quantity and function of other gut microbiota by affecting the level of *Lactobacillus*.

*Ruminococcus* is one of the earliest discovered gastric bacteria. It can degrade cellulose in the digestive tract and ferment glucose and xylose. Therefore, *Ruminococcus* can effectively break down the cell wall of plants in the digestive tract, which helps to stabilize the intestinal barrier and improve the body’s immunity [47]. *Ruminococcus* can induce anti-inflammatory or pro-inflammatory responses in the host, reflecting the characteristics of strain-specific immune regulation [48]. In this study, the relative abundance of *Ruminococcus_1* and *Ruminococcaceae_UGG-005* in the SD group is higher at the genus level, and the dominant bacteria in the SD group all belong to *Ruminococcus*, indicating that exposure of Se deficiency causes the imbalance of gut microbiota in rats, and the number and proportion of *Ruminococcus* changed significantly.

The characteristic pathological changes in KBD are necrosis of deep chondrocytes in growth plate cartilage and articular cartilage. Previous studies have shown that Se supplementation has a protective effect on growth plate cartilage, alleviates the necrosis of chondrocytes in growth plate. T-2 toxins can induce oxidative stress and reduce collagen type II and chondroitin sulfate in chondrocytes; therefore, Se deficiency and T-2 toxin were considered as most important risk factor for causing and development of KBD [49]. The human gut microbiome is strongly influenced by what they have taken in, such as Se deficiency and T-2 toxin, for example, Se deficiency affects the composition and colonization of the microbiome, which may interfere with the diversity of the microbiome [50,51]. And dietary selenium supplementation in mice can optimize the composition of gut microbiome and reduce the dysfunction of gut microbiome caused by low selenium [52]. In addition, the intestinal tract plays an important role in the metabolism and absorption of T-2 toxin, leading to intestinal mucosal damage, inflammation and oxidative stress, such as necrotizing enteritis and colibacillosis in animals. Fusarium toxins, such as T-2 toxin, was stable in gastrointestinal digestive fluid and cannot be absorbed by intestinal epithelial cells. When they come into contact with the gut microbiota, they can be efficiently hydrolyzed, allowing the metabolites to be easily absorbed from the gut [27]. According to our previous study on KBD patients, we found considerable variability in the gut microbiota, such as an increase in *Bacteroides* and a decrease in *Firmicutes*, which were similar to those in this study. At present, the mechanism of Se deficiency and fusarium toxin causing KBD has not been fully clarified. Based on the results of this study and the above discussion, we speculated that Se deficiency and T-2 toxin would affect the composition of human gut microbiota and its metabolites through dietary intake and intestinal absorption, resulting in the dysregulation of gut microbiota, and then induced joint cartilage damage through the cartilage–gut–microbiome axis, thereby triggering KBD. We believe that Se deficiency and T-2 toxin cause gut microbial dysregulation, which leads to joint damage, is one of the mechanisms triggering KBD. However, the specific mechanism is still unclear and needs further study.

## 5. Conclusions

In conclusion, this study comparatively analyzed the different compositions of gut microbiota in rats induced by Se deficiency and T-2 toxin, and identified and revealed the changes in the phylum, genus and species levels of gut microbiota. The results not only comprehensively reflect the situation of gut microbiota in rats with Se deficiency and T-2 toxin exposure, but also provide some clues for the role of the “cartilage–gut–microbiome” axis in the occurrence and development of KBD, and provide a scientific basis for promoting the study of the etiology and pathogenesis of KBD.

## Figures and Tables

**Table 1:** The relative abundance of species in the SD and T-2 groups at the phylum and genus level.

	SD	T-2
**Phylum**		
*Firmicutes*	81.39%	77.06%
*Bacteroidetes*	11.11%	14.85%
*Proteobacteria*	4.17%	3.95%
*Actinobacteria*	0.70%	0.50%
*Verrucomicrobia*	0.36%	1.81%
**Genus**		
*Ruminococcus_1*	12.62%	4.58%
*Ruminococcaceae_UCG-005*	10.31%	10.75%
*Ruminiclostridium_9*	8.26%	4.74%
*Lactobacillus*	2.82%	11.71%
*Muribaculaceae_unclassified*	5.16%	9.26%
*Firmicutes_unclassified*	7.55%	8.95%

**Phylum** means the relative abundance of species in the SD and T-2 groups at the phylum level. **Genus** means the relative abundance of species in the SD and T-2 groups at the genus level.

**Table 2:** Comparison of the differences in the microbiota between SD group and T-2 group at the genus level.

	SD	T-2
(A) *		
*Weissella*	Up (3.45)	up (3.06)
*Acetivibrio*	up (2.89)	up (5.30)
*Pseudoflavonifractor*	up (3.49)	up (4.61)
*Acetanaerobacterium*	down (−2.93)	down (−Inf)
(B) **		
	*Morganella* (Inf)	*Veillonella* (2.03)
	*Globicatella* (2.46)	*Ruminococcaceae_UCG-011* (Inf)
	*Duncaniella* (Inf)	*Eubacterium_xylanophilum_group* (1.98)
	*Oscillospira* (3.05)	*Phascolarctobacterium* (0.49)
(C) ***		
	*Lachnoanaerobaculum* (−4.49)	*Hydrogenoanaerobacterium* (-Inf)
	*Barnesiellaceae_unclassified* (−3.11)	*Rikenella* (−3.98)
	*Lachnoclostridium* (−1.44)	*Phocea* (−5.03)
		*Aerococcus* (−4.86)
		*DTU014_unclassified* (−3.49)
		*Erysipelatoclostridium* (−1.69)
		*Faecalitalea* (−3.30)

* Means the same significantly different species and abundance value (Log_2_FC). ** Means the significantly different species and abundance value (Log_2_FC) with increased levels. *** Means the significantly different species and abundance value (Log_2_FC) with decreased levels.

## Data Availability

All data generated or used during this study are available from the corresponding author and first author upon reasonable request.

## Supplementary Materials

The following supporting information can be downloaded at: https://www.mdpi.com/article/10.3390/nu15245027/s1, Figure S1: (a) the weighted PCoA results of the gut microbiota for the SD and NC groups; (b) Species correlation network in the SD group; Figure S2: COG database function annotation results between the SD and NC groups; Figure S3: (a) the weighted PCoA results of the gut microbiota for the T-2 and NC groups; (b) Analysis of Species significant differences at the genus level; (c) Species correlation network in the T-2 group; Figure S4: COG database function annotation results between the T-2 and NC groups.
