# Supplementary material for: Comparative Analysis of Gut Microbiota from Rats Induced by Se Deficiency and T-2 Toxin
Source: Nutrients. 2023 Dec 7;15(24):5027. doi: 10.3390/nu15245027 (PMC10745411; doi:10.3390/nu15245027)
Supplement: Supplementary file 1 [file nutrients-15-05027-s001.zip › Supplementary figure legends.pdf]

Supplementary figure legends:

Figure S1. (**a**) Principal coordinate analysis (PCoA) of the gut microbiota based on the weighted (ANOSIM, *P*= 0.876) UniFrac distance matrices for the SD and NC groups; (**b**) Species correlation network in the SD group. The dots in the figure indicate the different dominant bacterial genera, and the solid and dashed lines indicate positive and negative correlations, respectively. The thicker the lines, the stronger the correlation between the genera.

Figure S2. COG database function annotation results between the SD and NC groups;

Figure S3. (**a**) Principal coordinate analysis (PCoA) of the gut microbiota based on the weighted (ANOSIM, *P*= 0.902) UniFrac distance matrices for the T-2 and NC groups; (**b**) Analysis of Species significant differences at the genus level; (**c**) Species correlation network in the T-2 group.

Figure S4. COG database function annotation results between the T-2 and NC groups.
