# Supplementary figures and images for: Comparative Analysis of Gut Microbiota from Rats Induced by Se Deficiency and T-2 Toxin
Source: Nutrients. 2023 Dec 7;15(24):5027. doi: 10.3390/nu15245027 (PMC10745411; doi:10.3390/nu15245027)

PCoA plot (  $P=0.876$  )

a

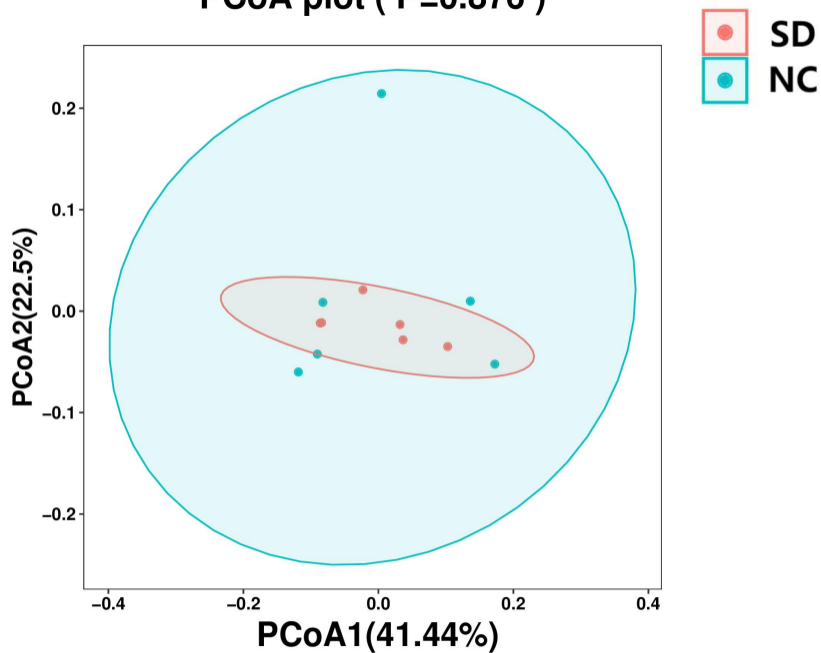

b

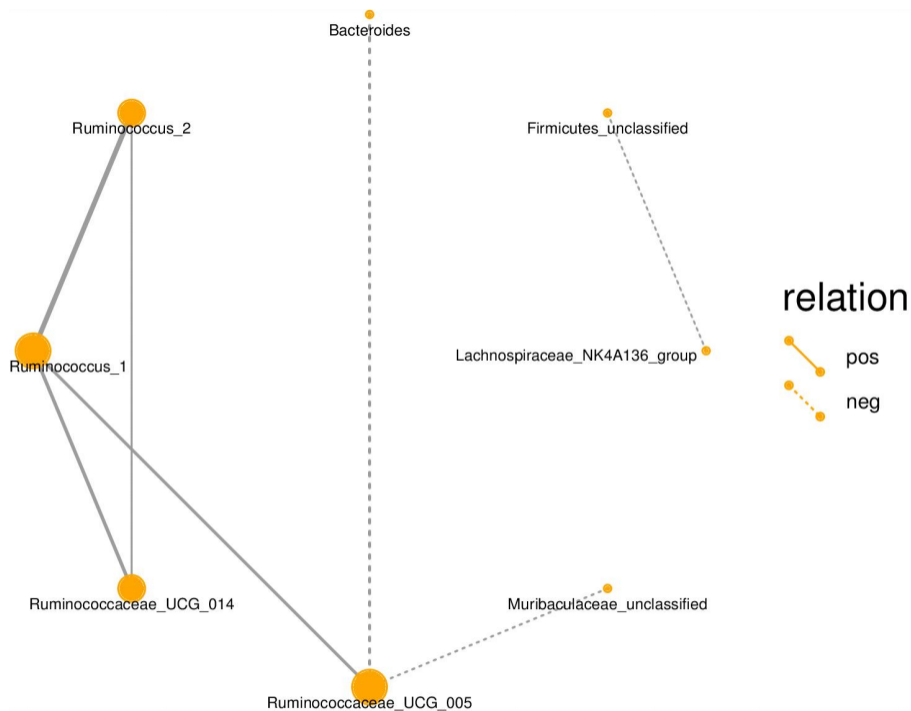

Supplement: Supplementary file 1 [file nutrients-15-05027-s001.zip › Figure supplementary S1.pdf]

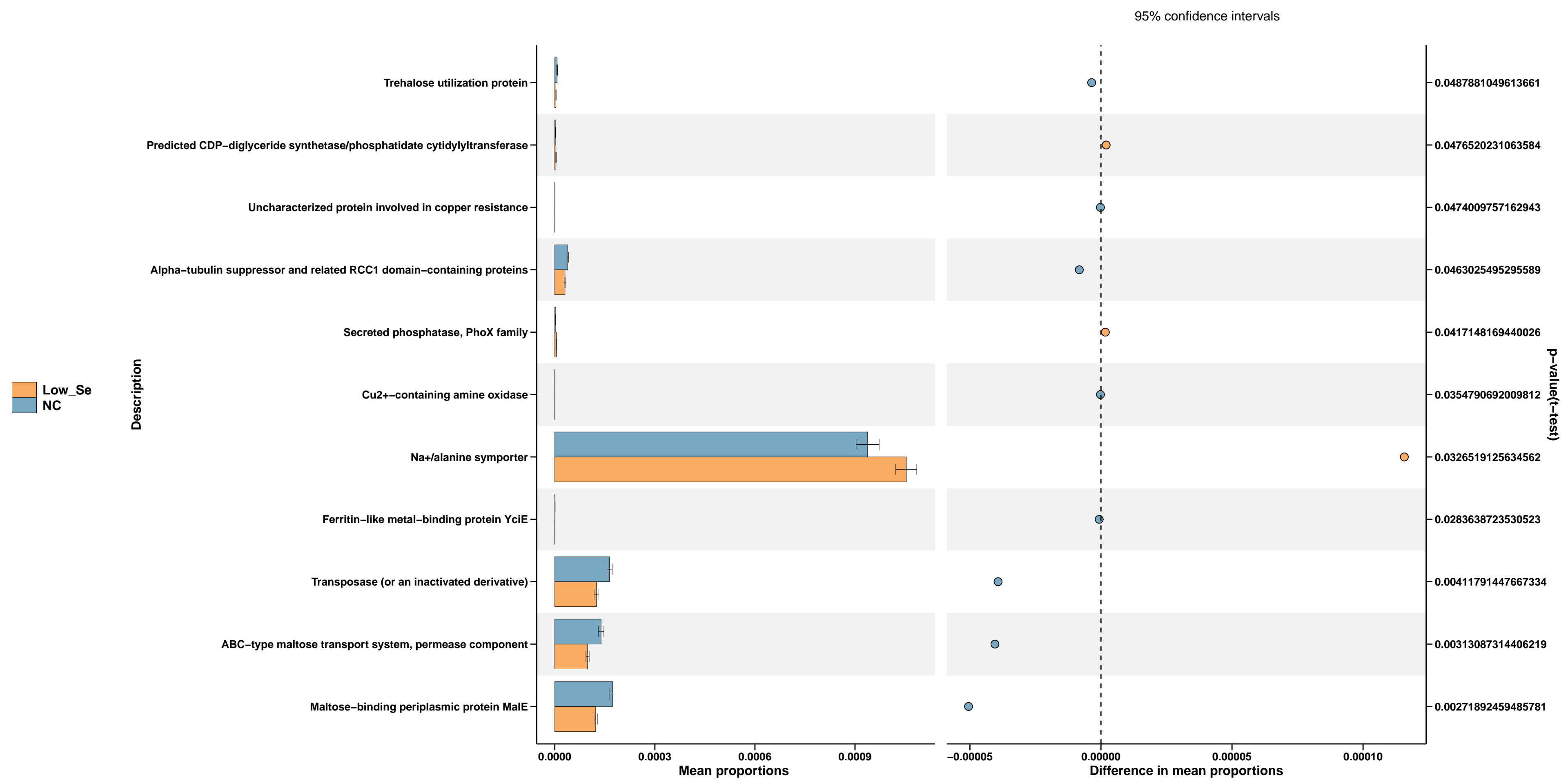

Supplement: Supplementary file 1 [file nutrients-15-05027-s001.zip › Figure supplementary S2.pdf]

a

PCoA plot ( P=0.902 )

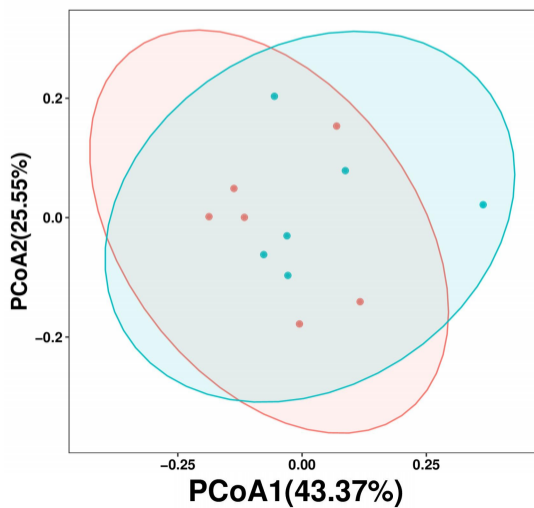

C

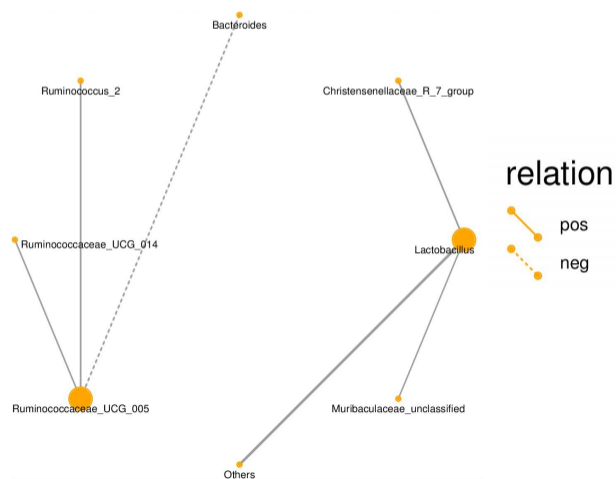

b

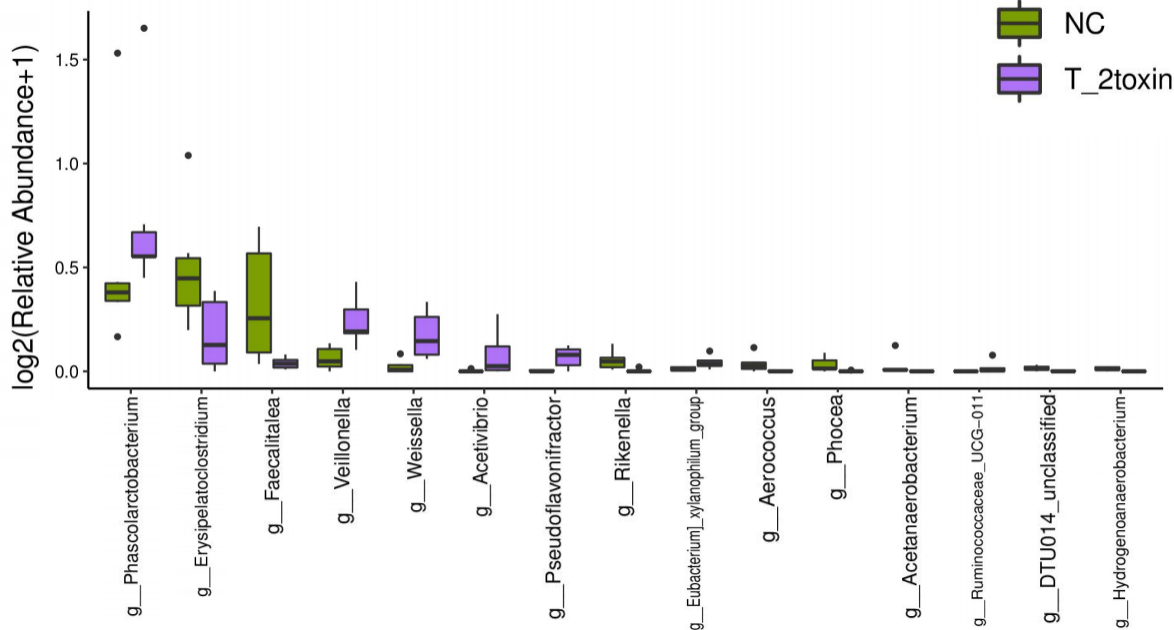

Supplement: Supplementary file 1 [file nutrients-15-05027-s001.zip › Figure supplementary S3.pdf]

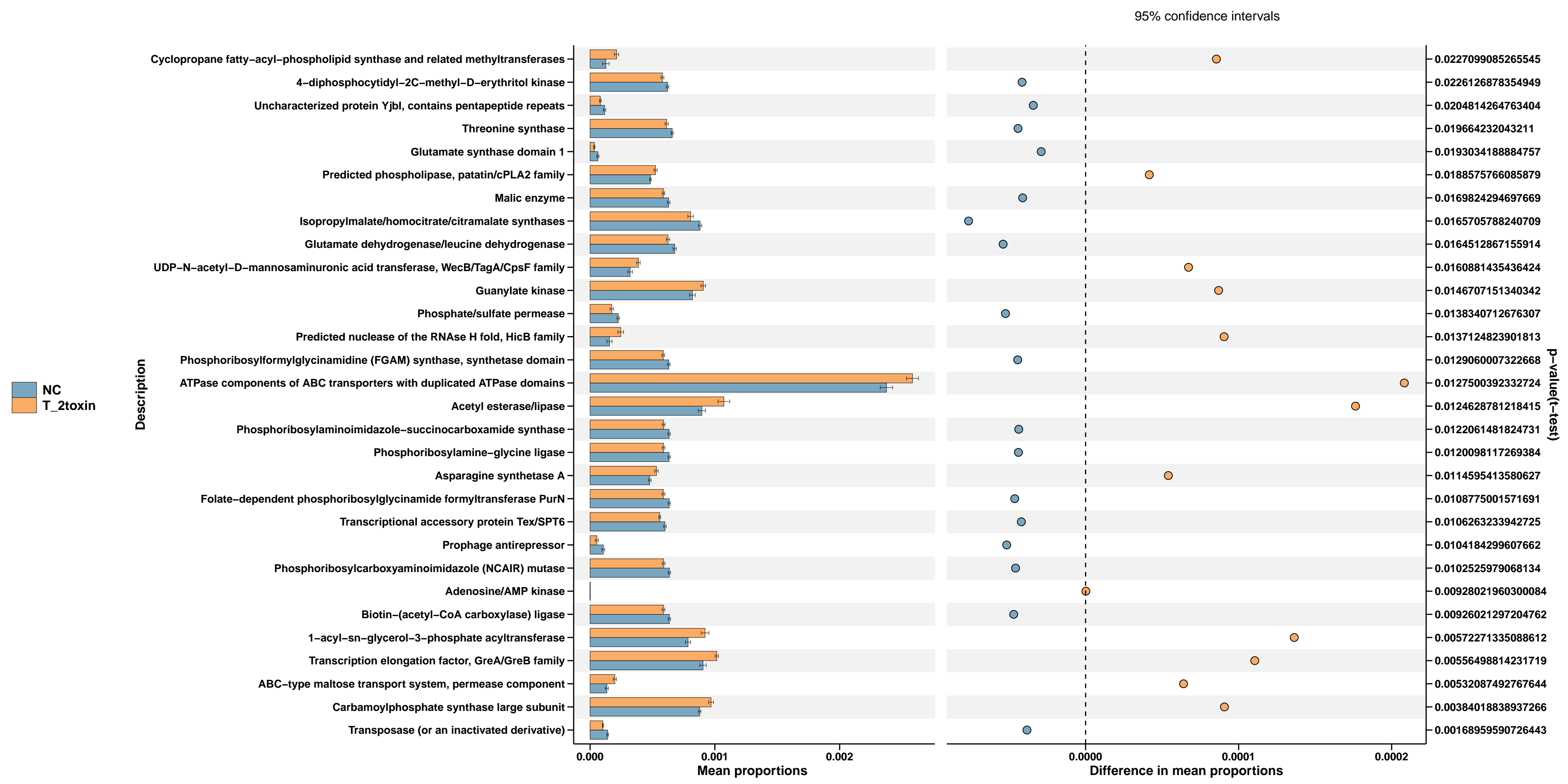

Supplement: Supplementary file 1 [file nutrients-15-05027-s001.zip › Figure supplementary S4.pdf]
